# A systematic review of adverse effects associated with systemic corticosteroids in the management of leprosy

**DOI:** 10.1371/journal.pntd.0014152

**Published:** 2026-03-26

**Authors:** Andie I. Lun, Barbara de Barros, Stephen L. Walker

**Affiliations:** 1 Department of Infection, University Hospitals Sussex NHS Foundation Trust, Royal Sussex County Hospital, Brighton, United Kingdom; 2 Department of Global Health and Infection, Brighton and Sussex Medical School, University of Brighton and University of Sussex, Brighton, United Kingdom; 3 The London School of Hygiene and Tropical Medicine, London, United Kingdom; 4 Hospital for Tropical Diseases, University College London Hospitals NHS Foundation Trust, London, United Kingdom; 5 Department of Dermatology, University College London Hospitals NHS Foundation Trust, London, United Kingdom; Marie Adelaide Leprosy Centre Pakistan, PAKISTAN

## Abstract

**Background:**

Leprosy is a chronic infectious disease caused by *Mycobacterium leprae* (*M. lepra*e*)* and *Mycobacterium lepromatosis* (*M. lepromatosis*), primarily affecting the skin and peripheral nervous system. Leprosy is complicated by immune-mediated reactions which are risk factors for nerve damage and disability. Systemic corticosteroids are the mainstay of therapy; however, prolonged use in chronic or recurrent reactions carries significant risks but the evidence on the short- and long-term adverse effects of corticosteroids in leprosy is limited. We conducted a systematic review to evaluate corticosteroid-associated adverse effects in leprosy affected individuals.

**Methodology:**

Eight electronic databases were searched (including PubMed, Embase and LILACS) without language or year restriction for studies reporting adverse effects associated with systemic corticosteroid use in individuals with leprosy. Eligible studies included randomised controlled trials, observational studies, case series and case reports. Data variability was assessed using Stata software.

**Main findings:**

A total of 111 studies were included; of which 22 were randomised controlled trials. Due to heterogeneity, findings were synthesised narratively. The most frequently reported adverse effects were metabolic complications, with approximately one-third of individuals developing corticosteroid-induced lipodystrophy. Infections were the second most common adverse effect (15.5%), followed by gastritis (12.6%). Infections accounted for three-quarters of corticosteroid-associated mortality, predominantly due to tuberculosis, with 88.2% of corticosteroid-associated mortalities occurring in individuals with erythema nodosum leprosum (ENL). There was an association between ENL and the development of cataract and osteoporosis, with 69.7% of cataract cases and 84.4% of osteoporosis cases occurring among individuals with ENL.

**Conclusion:**

This systematic review illustrates the range and severity of adverse effects affecting individuals with leprosy who received systemic corticosteroids. Although this review is limited by study heterogeneity, publication bias, and the scarcity of long-term data, it highlights the need for corticosteroid stewardship, structured pharmacovigilance and further research for safer therapeutic alternatives to corticosteroids for leprosy reactions.

## Author summary

Leprosy is a neglected tropical disease that causes stigmatising disability and is associated with significant reduction in quality of life. The infectious organism and the body’s immune response to it causes nerve damage often with severe, painful episodes called leprosy reactions. Leprosy reactions are the major risk factor for disability and are treated with corticosteroids. Corticosteroids are used in many inflammatory conditions with well recognised adverse effects, but data on these adverse effects in individuals with leprosy are limited.

We conducted a systematic review to characterise the nature and frequency of the adverse effects associated with corticosteroid use in people with leprosy. Our review found that adverse effects affected many organ systems and some were particularly frequent and severe in individuals who required prolonged treatment. Our results highlight the importance of monitoring patients with leprosy who are prescribed corticosteroids and reveal significant gaps within existing literature to inform future research. Strengthening pharmacovigilance of corticosteroid use and monitoring practices could help improve care.

## Introduction

Leprosy is a neglected tropical disease caused by *Mycobacterium leprae* (*M. leprae*) and *Mycobacterium lepromatosis* (*M. lepromatosis*) [1]. They are obligate intracellular, non-motile, acid-fast bacilli that have tropism for dermal macrophages, neuronal dendritic and Schwann cells [1,2]. A total of 172,717 new cases from 129 countries were reported to the World Health Organization (WHO) in 2024, among which 5.3% reported severe impairment with Grade 2 disability at diagnosis [3].

The clinical spectrum of leprosy is influenced by the affected individual’s immune response to the infection, specifically one’s cell-mediated immunity involving T cells and macrophages [4]. The Ridley and Jopling classification uses clinico-pathological correlation to classify leprosy into tuberculoid (TT) leprosy, borderline tuberculoid (BT), borderline borderline (BB), borderline lepromatous (BL) and lepromatous leprosy (LL) [5]. Individuals with TT leprosy have high cell-mediated activity, no identifiable bacteria in tissues and few skin lesions whereas people with LL have low cell-mediated activity, large numbers of bacteria and many skin lesions and/or widespread skin infiltration [4].

Leprosy reactions are immune-mediated responses to *M. leprae/lepromatosis* infection. A large proportion of individuals affected by leprosy will experience leprosy reactions. In a Brazilian randomised controlled trial (RCT) of anti-microbial treatment for WHO multibacillary leprosy, over 60% of participants experienced a leprosy reaction during six years of observation [6].

Leprosy reactions are the main risk factor for disability. The odds of disability are increased up to 12 times in individuals with LL [2,7]. Disability is associated with physical, psychological, economic and social impairments with reduced quality of life [8]. Leprosy reactions are categorized as Type 1 reactions (T1Rs), also known as reversal reactions, and erythema nodosum leprosum (ENL) or Type 2 reactions [9]. Reactions may occur before, during or after successful completion of antimicrobial therapy [2].

T1R may affect individuals with any type of leprosy and occur in 20–40% of individuals with borderline forms of leprosy [10–12]. T1R may present with erythematous skin lesions, oedema, neuritis (characterized by nerve tenderness) and nerve function impairment (NFI) [13,14]. NFI may be permanent if treatment is delayed, and 8–20% of individuals with NFI do not improve despite treatment [15,16]. ENL is a multisystem inflammatory disorder affecting individuals with BL leprosy or LL [17,18]. A single acute episode of ENL is rare, and most individuals experience recurrent or chronic episodes over many years [19,20]. A retrospective study of patients with ENL reviewed at The Hospital for Tropical Diseases in London found a median disease duration of 5 years [21]. In addition, a large retrospective cohort study conducted in India reported 80% of patients with ENL became dependent on corticosteroids [20]. Although ENL typically occurs during anti-microbial treatment, reactions may also occur up to eight years after treatment completion, further illustrating its unpredictable and chronic nature [22,23]. The clinical and public health burden of ENL is substantial, with a study conducted in Ethiopia reporting a 6.4 times higher odds of mortality in people with ENL compared to those with T1R [24].

Corticosteroids have been used to treat leprosy reactions and NFI since 1952 and have broad anti-inflammatory and immunosuppressive effects [25,26]. The WHO recommend oral corticosteroids for T1R and neuritis unresponsive to simple analgesia, starting with 0.5-1mg/kg/day tapering over 20 weeks [27]. Corticosteroids are recommended as the first line treatment for ENL using initial doses of prednisolone 30–40 mg/day, whereas recurrent or chronic T2R is best controlled with thalidomide at 100–400 mg/day [27]. Thalidomide is effective in ENL but is unavailable in many leprosy endemic countries due to its teratogenicity [28,29].

Corticosteroids are associated with a wide variety of adverse effects when used to treat inflammatory conditions. Adverse effects include infections, gastrointestinal symptoms, osteoporosis, fractures, glucocorticoid-induced Cushing syndrome (GICS), glucocorticoid-induced diabetes mellitus (GIDM), as well as cardiovascular disease and psychiatric complications [30–35]. GICS is characterised by corticosteroid-induced lipodystrophy, weight gain and acneiform eruption [36,37]. Adverse effects from corticosteroids are closely associated with dose and duration, with effects such as osteoporosis and GIDM arising early in treatment compared to cataracts and fractures which occur later [38]. Evidence from a meta-analysis found higher cumulative corticosteroid exposure to be associated with an increased risk of fracture [39], and a case-control study of participants with systemic lupus erythematosus found 12% increased odds of infection with each 1mg/day increase in prednisolone dose [40].

The frequency of leprosy reactions and lack of available alternatives to corticosteroids means many individuals are prescribed corticosteroids at high doses and for prolonged periods, exposing them to the risk of corticosteroid-associated adverse effects [20,24]. We wished to conduct a systematic review to evaluate the nature and frequency of AEs associated with corticosteroid in people affected by leprosy.

## Methods

The study was registered prospectively on PROSPERO on 27^th^ June 2025 (registration number CRD420251059485). The protocol is available at https://www.crd.york.ac.uk/PROSPERO/view/CRD420251059485.

This systematic review was conducted and reported following the Preferred Reporting Items for Systematic Reviews and Meta-Analyses (PRISMA) 2020 guidelines (S1 File).

### Search strategy

A comprehensive search was performed on 28^th^ May 2025, across eight major databases: PUBMED, MEDLINE, EMBASE (via OVID), The Cochrane Library (CENTRAL), CINAHL, LILACS, Global Index Medicus, and SCOPUS. The strategy combined controlled vocabulary terms (e.g., Leprosy, Steroids, Drug-related Side Effects and Adverse Reactions) with free-text keywords for corticosteroids, adverse outcomes, and leprosy reactions (including erythema nodosum leprosum, reversal reactions, Type 1 and Type 2 reaction). Boolean and proximity operators were used (S1 Text). A filter from Canada’s Drug Agency for adverse effects was consulted to supplement the search strategy [41].

### Inclusion and exclusion criteria

RCTs, prospective and retrospective cohort studies, case-control studies, case series, and case reports that met pre-specified inclusion criteria were included. Non-human studies and in-vitro investigations were excluded. No language or temporal restrictions were imposed; articles not in English were translated using Google Translate. Letters to the editor, editorials, and commentaries were excluded unless they reported original outcome data. Studies were eligible if they included individuals with leprosy, diagnosed by WHO 2018 criteria or laboratory confirmation [42], who received systemic corticosteroids for nerve function impairment or leprosy reactions. Comparators included placebo, other medications, or different corticosteroids regimens. Outcomes of interest were corticosteroid-related adverse effects.

Backward citation searching was conducted by reviewing the reference lists of all included studies, and authors were contacted for further information if required. Conference proceedings were included as grey literature. The study selection process was conducted by one reviewer, with any uncertainties resolved through discussion with a second reviewer and, if necessary, adjudicated by a third reviewer.

### Data extraction

Data from eligible studies were extracted using a standardized Microsoft Excel spreadsheet. The extracted data included study design, population characteristics, indication, intervention details (type, dosage, comparator(s), placebo, alternative medicines, or dosage variations), and all reported AEs associated with systemic corticosteroid therapy. When necessary, study authors were contacted to clarify or provide missing information to ensure data completeness.

The primary outcome of interest was AEs associated with corticosteroid use for the management of leprosy reactions. ‘Adverse effect’ was defined according to the pharmacovigilance body of WHO, as “a negative or harmful patient outcome that seems to be associated with treatment, including there being no effect at all” [43].

Corticosteroid exposure was categorized as either ‘corticosteroid-naïve,’ defined as no prior exposure to corticosteroid therapy, or ‘corticosteroid-experienced,’ defined as previous exposure or established tolerance to corticosteroid therapy.

### Risk of bias assessment

All included studies were critically appraised. Version 2 of the Cochrane risk-of-bias tool (RoB 2) was used to critically appraise RCTs [44]; the Newcastle Ottawa Scale was used to assess prospective and retrospective cohort studies [45]; the Joanna Briggs Institute critical appraisal tool was used to assess case series and case reports [46].

### Statistical analysis

Statistical analyses were performed using StataCorp (version 18). The extent of heterogeneity among included studies was determined by calculating the I² statistic and conducting tests of homogeneity. In instances where there were zero numerator events in outcome data, reciprocal correction was applied following recommendations in the Cochrane Handbook [47]. Risk ratio (RR) and 95% confidence interval (CI) were calculated where possible when analysing data from narrative synthesis. Where only total AEs were reported (allowing for multiple events per individual), the event risk per person was used to avoid violating assumptions of independence [48].

### Ethical approval

This study was undertaken as part of the requirements of a master’s degree at the London School of Hygiene & Tropical Medicine. Ethical approval for this study was requested through submission of a combined academic, risk assessment and ethics form, which was reviewed by the Research Governance & Integrity Office as not requiring ethical approval from the ethics committee.

## Results

### Overview of all included studies

A total of 111 studies from 19 countries published between 1955 and 2025 were included. Most were from India (n = 50) and Brazil (n = 15), which reflects the current global distribution of leprosy. The review identified 22 RCTs, 14 prospective studies, 5 retrospective studies, 56 case reports and 14 case series. Eight information requests were sent to authors, or to libraries when author contact details were unavailable; none received a response. Two supplementary publications were used to extract AE data for the TRIPOD and TENLEP trials [49,50], as some AEs were reported separately from the primary trial publications.

**Fig 1:**
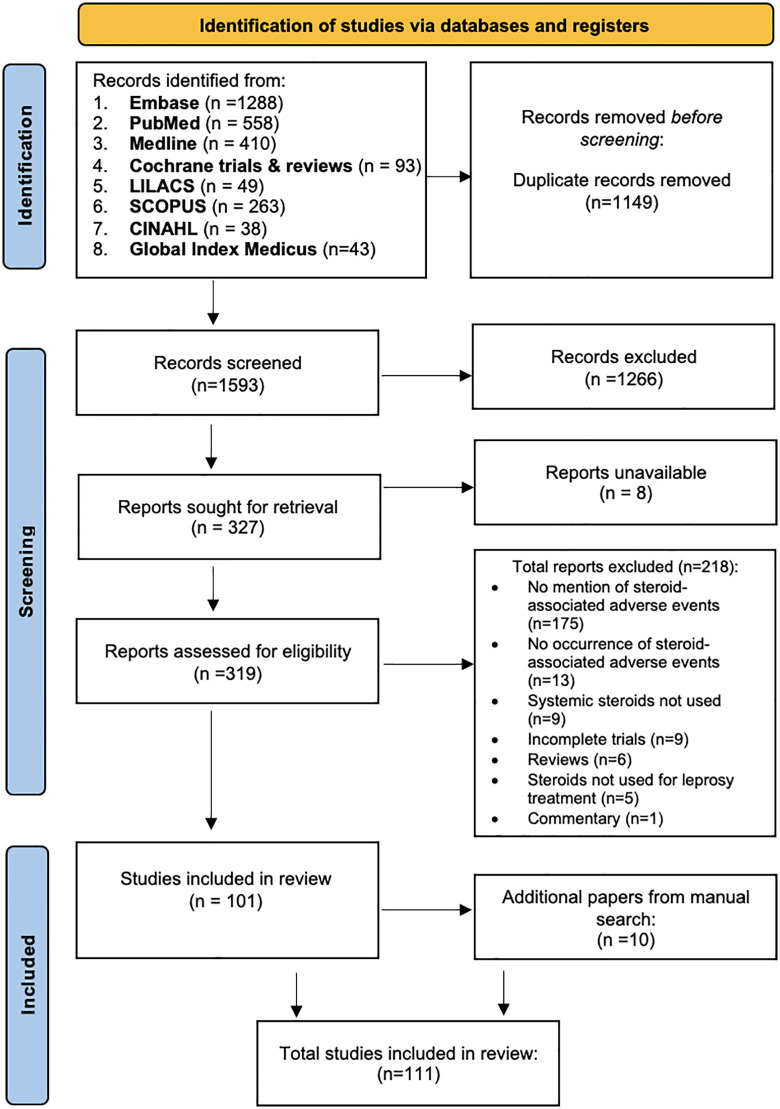
Preferred Reporting Items for Systematic Reviews and Meta-Analyses (PRISMA) flow diagram for the different stages of the systematic review.

Prednisolone was the most frequently used corticosteroid, with cortisone appearing in earlier studies. Participants were aged 3–80 years old though mostly adults; 76.1% were male and 62.1% of studies focused on ENL. The results of study identification and screening are presented in a flow diagram as shown in Fig 1 [51].

### Randomised controlled trials

A total of 22 RCTs were categorized according to reaction type: prophylaxis (n = 2), T1R and NFI (n = 11), and ENL (n = 9). The RCTs compared corticosteroid with placebo, corticosteroid with non-corticosteroid agents and lower corticosteroid doses with higher ones. Trials with identical corticosteroid regimens or unquantifiable data were excluded from meta-analysis.

### Randomised controlled trials of Type 1 reactions and nerve function impairment

Table 1 summarises the characteristics of the included RCTs on T1R and NFI. All were double blinded except one. The TRIPOD trials tested prevention (TRIPOD 1), early treatment (TRIPOD 2) and late treatment (TRIPOD 3) of NFI in leprosy [15,51,52]. The TENLEP trials compared prednisolone durations for recent NFI (TENLEP clinical) and examined early treatment of subclinical neuropathy (TENLEP subclinical) [53]. Of 1,920 participants, 86% received oral prednisolone and one study used pulsed intravenous methylprednisolone [54]. The corticosteroid treatment duration ranged from 16 to 20 weeks, with follow-up of up to 19.5 months. No trial was at high risk of bias. The details of risk of bias of RCTs can be found in S1 Table.

**Table 1:** Summary of randomised controlled trials reporting corticosteroid-associated adverse effects in Type 1 reaction & nerve function impairment.

Study ID	Study Type	Sample size	Intervention (A)	Comparator (B)	No. of patients (A: B) [Male %]	Duration of therapy (A: B); Duration of follow-up	No. of adverse effects (A: B)	Risk ratio (95% CI)	*p*-value	Risk of Bias (ROB2)
Corticosteroid vs Placebo										
TENLEP sub-clinical [50]	Double blind RCT	364	PO Prednisolone 1mg/kg/day (tapered)	Placebo	181: 182 [N/A]	20 weeks; 18 months	105:76	1.39 (1.13 – 1.72)	0.002	Assessment not possible †
Van Brakel et al 2003 (TRIPOD 2) [52] *	Double blind RCT	75	PO Prednisolone 40mg/day (tapered)	Placebo	41:34 [73.0%]	16 weeks; 12 months	13:11	1.20 (0.57 – 2.52)	0.640	Low
Richardus et al 2003a (TRIPOD 3) [15] ‡	Double blind RCT	92	PO Prednisolone 40mg/day (tapered)	Placebo	40:52 [78.9%]	16 weeks; 12 months	2:3	0.86 (0.15 – 4.94)	0.872	Low
Higher total corticosteroid dose vs Lower total corticosteroid dose										
Garbino et al 2008 [55]	Double blind RCT	21	PO Prednisolone 2mg/kg/day	PO Prednisolone 1mg/kg/day	12:9 [81.0%]	24 weeks; 6 months	2:0	19.07 (0.020-18593)❡	0.400	Some
Marlowe et al 2004 [56]	Open label RCT	40	PO Prednisolone 40mg/day (tapered)	PO Prednisolone 40mg/day (tapered) + PO Azathioprine	20:20 [87.5%]	12: 8 weeks; 6 months	2:1	2.00 (0.20 – 20.3)	0.586	Some
Wagenaar et al 2017 (TENLEP clinical) [53]	Double blind RCT	868	PO Prednisolone 1mg/kg/day (32 weeks)	PO Prednisolone 1mg/kg/day (20 weeks)	439:429 [76.6%]	32:20 weeks; 19.5 months	312:288	1.06 (0.97 – 1.16)	0.209	Low
Walker et al 2011 [54]	Double blind RCT	42	1g intravenous methylprednisolone x 3 days + PO prednisolone 40mg/day (tapered)	PO Prednisolone 40mg/day (tapered)	20:22 [78.6%]	16 weeks; 11 months	11:12	1.01 (0.58 – 1.75)	0.976	Low
Corticosteroid vs Corticosteroid + Adjunct										
Lambert et al 2016b [16]§	Double blind RCT	73	PO Prednisolone 40mg/day (tapered)	PO Prednisolone 40mg/day (tapered) + PO Ciclosporin	38:35 [79.5%]	20:4 weeks; 8 months	N/A	N/A	N/A	Low
Lockwood et al 2017 [57]	Double blind RCT	345	PO Prednisolone 40mg/day (tapered)	PO Prednisolone 40mg/day (tapered) + PO Azathioprine of varying duration	87:258 [86.1%]	20 weeks; 12 months	N/A	N/A	N/A	Low

* Minor and major effects from TRIPOD 2 calculated based on supplementary data from Richardus et al 2016b

† Paper currently still in preparation

‡ Only major effects reported to have occurred in TRIPOD 3

§ Proportion of patients with adverse effects unreported

❡ Reciprocal correction applied

PO = Per orum

N/A= Not available

ROB2 = The Cochrane Risk of Bias 2 tool

Most participants were male (89.2%), though data other than corticosteroid-associated AEs for the TENLEP subclinical trial were not reported [50]. Placebo-controlled and dose-comparison trials showed no difference in overall AE risk with prednisolone.

Three RCTs directly compared oral prednisolone with placebo and showed negligible heterogeneity (I² < 0.001; Q = 1.53, *p* = 0.470) [15,50,52]. Data extraction was limited by pooled reporting and incomplete data [15,50,52].

### Randomised controlled trials of erythema nodosum leprosum

Between 1969 and 2025, 11 RCTs investigated and reported adverse effects associated with corticosteroid therapy for ENL (Table 2). Prednisolone was the most frequently used, given at doses between 15 and 60mg daily for 4 weeks to 6 months; betamethasone was used in one study [58]. Three trials were open-label and two reported the occurrence of AEs but not their frequency [58,59]. Overall, 288 of 468 (61.5%) participants received corticosteroids, most were male (82.7%), and the mean follow-up was 6.5 months.

**Table 2:** Summary of randomised controlled trials reporting corticosteroids-associated adverse effects in Erythema Nodosum Leprosum.

Study ID	Study Type	Sample size	Intervention (A)	Comparator (B)		No. of patients (A: B) [Male %]	Duration of therapy; Duration of follow- up	Adverse effects (A: B)	Risk ratio (95% CI)	*p*-value	Risk of Bias (ROB2)
Corticosteroid vs Non-corticosteroid medication											
Karat et al 1969 [62]*	Double blind RCT	50	PO Prednisolone 15mg/day (tapered)	Indomethacin; Chloroquine; Aspirin		13:37 [N/A]	4 weeks; 6 months	9:18	1.42 (0.87- 2.32)	0.200	Some
Kar et al 2015 [63]†	Open label RCT	66	PO Prednisolone 1mg/kg/day (tapered)	Thalidomide		17:16 [92.4%]	20 weeks; 6 months	9:8	1.20 (0.63-2.30)	0.576	High
Karat et al 1970 [60]	Double blind RCT	24	PO Prednisolone 30mg (tapered)	Clofazimine		12:12 [N/A]	12 weeks; [N/A]	6:12	0.50 (0.28-0.88)	0.300	Low
Ing et al 1969 [61]	Double blind RCT	30	PO Prednisolone 15mg/day (tapered)	Indomethacin		14:16 [N/A]	4 weeks; 1 month	1:0	0.13 (0.02-0.88)	0.004	Some
Kaur et al 2009 [64]‡	Open label RCT	60	PO Prednisolone 40mg/day (tapered)	Thalidomide		30:30 [81.7%]	8 weeks; 12 months	N/A	N/A	N/A	Some
Corticosteroid vs Corticosteroid + Adjunct											
Lambert et al 2016a [66]	Double blind RCT	33	PO Prednisolone 60mg/day (tapered)	PO Prednisolone 40mg/day (tapered) + PO Ciclosporin		16:17 [78.8%]	16 weeks; 8 months	16:17	1.00	–	Low
Hanumanthu et al 2021 [67]§	Double blind RCT	57	PO Prednisolone 40mg/day (tapered)			29:28	12 weeks	N/A	N/A	N/A	Some
			Minocycline	Clofazimine							
Roy et al 2015 [68]§	Double blind RCT	20	PO Prednisolone 40mg/day			10:10 [82.5%]	12 weeks	N/A	N/A	N/A	Low
			Pentoxifylline	Clofazimine							
Martinus et al 2020 [69] §	Double blind RCT	29	PO Methylprednisolone (equivalent to 40mg/day prednisolone tapered)			15:13 [85%] [78.6%]	12 weeks	N/A	N/A	N/A	Low
			Placebo	Thalidomide							
Unquantifiable AEs											
Sakhare et al 2024 [59]	Open label RCT	30	PO Prednisolone 80 mg/day (tapered)			10:10:10 [Unavailable]	12 weeks	N/A	N/A	N/A	High
			Clofazimine	Methotrexate	Sodium stibogluconate						
Girdhar et al 2002 [58]	Open label pilot study	9	PO Betamethasone in 5% dextrose 40mg for 3 days every 4 weeks for 6 months	Placebo		4:5 [Unavailable]	6 months; 6 months	N/A	N/A	N/A	High

* Study had 4 arms: Prednisolone, Indomethacin, Chloroquine and Aspirin; non-corticosteroid medications were combined as one group

† Study had 4 arms: Prednisolone only; Thalidomide only; Thalidomide + Prednisolone; Clofazimine + Prednisolone. Prednisolone-only and thalidomide-only groups used for risk ratio

‡ Proportion of patients with adverse effects not reported

§ Identical corticosteroid regime in both arms

N/A = Not available

PO= Per orum

CI = Confidence Interval

Five RCTs compared prednisolone with non-corticosteroid medications, but one was excluded from meta-analysis due to insufficient AE data [60–64]. Analysis of the remaining four showed moderate heterogeneity (I² = 47.5%, Q = 4.12, *p =* 0.128). Given the small number of trials, modest sample sizes and only one study with low risk of bias, meta-analysis was deemed inappropriate (S1 Table). The risk ratio of the four RCTs ranged from 0.02 - 2.32, with no studies demonstrating a significant increase in risk of AEs corticosteroids compared with non-corticosteroid regimens (Table 2).

### Randomised controlled trials of corticosteroids for leprosy reaction prophylaxis

Two double-blind RCTs assessed corticosteroids for preventing leprosy reactions and NFI (Table 3). Doull et al compared four interventions (placebo, dexamethasone 1.5mg/day, methandrostelone and mefenamic acid) [65]. There was no significant difference in the risk of AEs between the placebo and corticosteroid arms (RR 2.76, 95% CI 0.11-66.90; *p* = 0.530), although there is potential bias from unclear randomisation and missing outcome data (S1 Table). Smith et al 2004 (TRIPOD 1), assessed oral prednisolone 20mg/day for 12 weeks versus placebo [51]. The study reported a 1.68 times higher risk in overall AEs in the corticosteroid arm compared with placebo (95% CI 1.23-2.36; *p* = 0.002) [51]. The overall risk of bias was low, supported by a large sample size and clear methods for randomisation and outcome assessment.

**Table 3:** Summary of leprosy reaction or nerve function impairment prophylaxis trials reporting corticosteroid-associated adverse effects.

Study ID	Study Type	Sample size	Intervention (A)	Comparator (B)	No. of patients (A:B)	Duration of therapy	Adverse Effects (A:B)	Risk ratio (95% CI)	*p*-value	Risk of bias
Doull et al 1967 [65]*	Double blind RCT	346	PO Dexamethasone 1.5mg/day	Placebo	88:81 [76.6%]	24 weeks	0:1	2.76 (0.11-66.90)	0.530	Some
Smith et al 2004 (TRIPOD 1) [51]	Double blind RCT	636	PO Prednisolone 20mg/day	Placebo	239:239 [70.3%]	3 months	73:45	1.68 (1.23-2.36)	0.002	Low

*Study had 4 arms: Placebo, Dexamethasone, Methandrostelone, Mefenamic acid. Risk ratio performed for placebo and dexamethasone groups only.

### Prospective cohort studies

Fourteen prospective cohort studies were included and had a mean follow-up of 29.6 months (S3 Table) [70–83]. Of the fourteen studies, ten included participants receiving corticosteroids for T1R, NFI or ENL, although three of these did not quantify AEs. Four of the fourteen studies assessed only individuals with ENL [77–79,84]. Two studies included pre-defined, non-randomised comparison groups [79,82], while the others were single-group prospective cohorts, generally with moderate risk of bias (S2 Table). Prednisolone was used in all but one study which used betamethasone, and prednisolone doses ranged from 25-60 mg/day for 3 weeks to 14 months [70]. Overall, studies including participants with T1R, NFI or ENL reported AE rates ranging from 1.0 to 213.3 per 100 patients, while studies restricted to ENL alone reported rates ranging from 16.7 to 101.2 per 100 patients (S3 Table).

### Retrospective studies

Five retrospective studies were included (S4 Table) [21,24,85–87]. Four of which studied individuals with ENL, whereas Siagan et al. included individuals with T1R or ENL [85]. Among included individuals, 68.7% were male, and concomitant therapies included thalidomide, clofazimine, azathioprine and chloroquine; two studies also involved methotrexate and ciclosporin [24]. Most studies were of moderate risk of bias due to a lack of a comparator arm (S2 Table).

#### Case series and case reports.

A total of 13 case series and 58 case reports were included, with 84.6% of case series involving individuals with ENL (S5 Table). The case reports described 61 patients, predominantly male (78.7%); 81% had prior corticosteroid exposure and 86.7% had ENL. The mean treatment duration was 13 months with a mean follow-up of 16 months. Infection was the most frequently reported AE, and two-thirds of corticosteroid-related deaths (66.7%) were reported in case reports, all in individuals with ENL.

### Adverse effects associated with corticosteroids

#### Mortality.

Seventeen corticosteroid-associated deaths were identified (Table 4). Infections accounted for 76.5% of cases, including 23.1% due to *Mycobacterium tuberculosis*. Two deaths resulted from septic shock secondary to *Strongyloides stercoralis* hyperinfection syndrome [88,89]. All deaths reported were in individuals with chronic, severe ENL, except two with undocumented reaction status. Two individuals had been self-administering corticosteroids unsupervised for >18 months [80,90].

**Table 4:** Table detailing reported cases of mortality associated with corticosteroid use.

Cause of death	Study ID	Age	Sex	Case definition	Steroid regimen	Description
Tuberculosis	Sugumaran 1998 [72]	Unknown	Unknown	Unknown	Unknown	Three patients were reported to have died from pulmonary tuberculosis
	Walker et al 2014 [24]	33	Male	ENL	PO Prednisolone 40mg/day	Death from pulmonary tuberculosis while self-medicating with prednisolone
	Mishra et al 2024 [80]	60	Female	BL with severe erythema necroticans	PO Prednisolone (self-administered) for 18 months	Developed cerebral tuberculoma and tubercular arteritis after prolonged self-medication with prednisolone, leading to stroke and death
Other bacterial infection	Davis et al 1998 [90]	45	Male	LL with severe ENL	Intravenous Hydrocortisone 100mg 8 hourly and injectable dexamethasone 4mg twice a day for a week + prednisolone and injectable beclomethasone intermittently for a ‘long time’	Cause of death unclear; patient took clofazimine 100 mg thrice daily for seven months alongside high-dose corticosteroid
	Walker et al 2014 [24]	25	Female	LL with chronic ENL	PO Prednisolone 55mg/day (duration N/A)	Died of pneumonia
	Walker et al 2014 [24]	20	Female	LL with chronic ENL	PO Prednisolone 60mg/day (duration N/A)	Died of septic shock
	Pai et al 2017 [91]	45	Female	Unclear	PO Prednisolone 30mg/day (duration N/A)	Death due to Klebsiella pneumonia with septic shock; patient also had glucocorticoid-induced diabetes
	Zhu et al 2017 [92]	24	Male	BL with recurrent, severe ENL	PO Methylprednisolone 60–80mg/day intermittently for 15 months	Died of meningitis with secondary *Pseudomonas aeruginosa* infection
*Strongyloides stercoralis* hyperinfection syndrome	Leang et al 2004 [88]	19	Male	LL with chronic ENL	PO Prednisolone 50mg/day for 6 months	Died of septic shock and acute respiratory distress from SHS
	Mutreja et al 2015 [89]	36	Male	LL with ENL	PO Prednisolone 50mg/day for 12 months	Adrenal crisis occurred as a complication of SHS and sepsis
Other infections	Walker et al 2014 [24]	22	Male	Bl with chronic ENL	PO Prednisolone (dose and duration N/A)	Died of multiorgan failure secondary to viral hepatitis
	Walker et al 2014 [24]	45	Female	BL with chronic ENL	Unknown	Died of cardiac arrest with concurrent herpes zoster infection
	Walker et al 2014 [24]	19	Female	Chronic ENL	Unknown	Died of sepsis with severe epistaxis
Other						
Haemorrhage shock	Sugumaran 1998 [72]	40	Male	ENL	PO Prednisolone 30mg/day for 1 week	Died of haemorrhagic shock in the context of alcoholism
Perforated gastric ulcer	Lambert et al 2016a [66]	Unclear	Unclear	ENL	Prolonged and intermittent course of prednisolone	Died of multiorgan failure secondary to perforated gastric ulcer
Diabetic Ketoacidosis (DKA)	Walker at al 2014 [24]	15	Male	ENL	PO Prednisolone for 2 months	Died of DKA with concurrent pulmonary tuberculosis
Unspecified	Walker et al 2014 [24]	28	Male	LL with chronic ENL	N/A	Died of multiorgan failure

N/A = Not available.

Metabolic effects were the most frequently reported followed by infection. Glucocorticoid-induced Cushing syndrome (GICS) occurred at a rate of 65.1 per 100 patients: 32.2% of individuals at risk developed facial corticosteroid-induced lipodystrophy (CIL), 16.8% had acneiform eruptions and 10.5% developed weight gain. 15.3% developed an infection, among those 63.3% were fungal.

### Metabolic and endocrine adverse effects

#### Glucocorticoid-induced cushing syndrome.

GICS was the most frequently reported AE. In RCTs of T1R and NFI, 26.8% of participants developed corticosteroid-induced lipodystrophy (CIL) and 25.1% acneiform eruption (Table 5). Placebo-controlled trials suggested increased risk of CIL and corticosteroid-related acneiform eruptions [50,51]. Smith et al reported a 9.31 times higher risk of developing acneiform eruptions in participants on corticosteroid therapy compared to placebo (RR = 9.31, 95% CI 1.19-73.1; *p* = 0.009), and the TENLEP subclinical trial found a 1.82 (95% CI 1.22-2.45; *p* = 0.016) and 1.73 (95% CI 1.29-22.37; *p* < 0.001) times increase in acne and CIL respectively among those on corticosteroids [50,51]. No dose-response association was shown in dosage-comparison trials [53,54].

**Table 5:** Adverse effects reported in randomised controlled trials for T1R & NFI.

Adverse effect	Proportion of participants with AE (%)*	Proportion of RCTs reporting AE (%)†	Range of proportion of participants with AE in RCTs (%)
Glucocorticoid-induced Cushing syndrome‡			
Corticosteroid-induced lipodystrophy	26.8	50.0	4.8 - 41.2
Acneiform eruption	25.1	37.5	17.8 - 74.7
Weight gain	23.9	25.0	15.5 - 41.1
Other§	9.0	1 RCT	–
Gastritis	22.4	75.0	2.5 - 45.1
Infection	11.3	62.5	7.2 - 35.7
Fungal	10.5	37.5	7.2 - 24.6
Bacterial	0.1	25.0	0.5 - 1.4
Other infections	11.3	37.5	2.5 - 79.5
Glucocorticoid-induced diabetes mellitus	2.2	75.0	0.7 - 7.0
Hypertension	1.2	62.5	0.9 - 5.5
Neuropsychiatric effects	0.8	37.5	0.2 - 6.8
Peptic ulceration	3 cases	25.0	0.2 - 2.4
Gastrointestinal bleed	2 cases	1 RCT	–
Cataracts	1 case	1 RCT	–
Osteoporosis	1 case	1 RCT	–

*Denominator = total number of participants at risk (n=1609)

†Denominator= total number of RCTs for T1R included in review (n=8)

‡Frequency of GICS could not be determined due to incomplete and overlapping reporting

§Non-specific GICS

In ENL, 13.5% developed acneiform eruptions, with up to 41.7% of participants developing CIL with 30mg prednisolone tapered over 8 weeks in an RCT [60] (Table 6). GICS AEs were reported only in individuals receiving corticosteroid. Lambert et al. reported higher risk of acne with 60mg prednisolone daily compared to prednisolone 40mg daily and ciclosporin (RR 3.61, 95% CI 0.92-14.1; *p* = 0.027) [66]. A mean of 61.3% of treated individuals developed CIL across six prospective cohorts, with onset typically within weeks and resolution after corticosteroid cessation. All individuals with GICS reported in case series had ENL. Despite the high prevalence of GICS across all study designs, no withdrawals from randomised controlled trials were reported, though visible changes can cause considerable distress [72].

**Table 6:** Adverse effects reported in randomised controlled trials for erythema nodosum leprosum.

Adverse effect	Proportion of participants with AE (%)*	Proportion of RCTs reporting AE (%)†	Range of proportion of participants with AE (%)
Gastritis	20.7	58.3	1.8 - 90.9
Glucocorticoid-induced Cushing syndrome‡			
Acneiform eruption	13.5	1 RCT	–
Corticosteroid-induced lipodystrophy	9.0	33.3	15.8 - 41.7
Weight gain	4.8	25.0	2.4 - 36.7
Other§	2.1	1 RCT	–
Infection	12.0	25.0	4.8 – 90.9
Fungal	5.4	16.7	2.4 - 51.5
Bacterial	2 cases	16.7	2.4 - 3.0
Other infections	9.3	16.7	2.4 - 69.7
Glucocorticoid-induced diabetes mellitus	3.9	33.3	6.1 - 10.0
Neuropsychiatric effects	2.3	25.0	1.8 - 12.1
Hypertension	1.8	25.0	3.0 - 10.0
Gastrointestinal bleed	2 cases	1 RCT	–
Peptic ulceration	1 case	1 RCT	–
Cataract	1 case	1 RCT	–
Glaucoma	1 case	1 RCT	–
Deep vein thrombosis	1 case	1 RCT	–

*Denominator= total number of participants at risk (n=333)

†Denominator= total number of RCTs for T2R included in review (n=12)

‡ Frequency of GICS could not be determined due to incomplete and overlapping reporting

§ Non-specific GICS

### Glucocorticoid-induced diabetes mellitus (GIDM)

In RCTs, GIDM occurred in 3.9% of participants with ENL compared to 2.2% in T1R (Table 5–6). No RCT showed a significant difference in prevalence of GIDM between individuals who received corticosteroid compared with those who received placebo. This may be due to small sample sizes and event rarity. Higher corticosteroid doses were not consistently associated with increased risk, and two small ENL RCTs reported GIDM, with cases managed using oral hypoglycaemic agents [16,53,63,64]. 45.4% of all RCTs did not report any monitoring of symptoms or signs of GIDM, and diagnostic methods, when reported, included urinary glucose, random plasma glucose and glycosylated haemoglobin levels (HbA1C). The median duration of follow-up in RCTs was 8.5 months.

Five prospective cohorts reported GIDM in 2.7-6.1% of participants, mostly within 3 months of initiation (Table 7). Two studies noted symptom resolution after cessation, and notably, Papang et al. found higher cumulative corticosteroid exposure strongly predicted GIDM [72,73,75].

**Table 7:** Adverse effects reported in prospective cohort studies.

Adverse effect	Proportion of participants with AE (%)*	Proportion of prospective cohort studies reporting AE (%)†	Range of proportion of participants with AE (%)
Glucocorticoid-induced Cushing syndrome			
Corticosteroid-induced lipodystrophy	61.3	35.7	11.1 - 100.0
Acneiform eruption	18.3	28.6	19.3 - 100.0
Weight gain	1.9	14.3	11.1 - 36.6
Others‡	0.3	14.3	2.9 – 13.3
Infection	11.5	50.0	2.2- 24.8
Fungal	13.6	21.4	11.1 - 22.9
Bacterial	1.0	28.6	1.1 - 22.2
Cataract	5.3	21.4	0.5 - 9.8
Gastritis	4.5	28.6	2.4 - 8.0
Glucocorticoid-induced diabetes mellitus	3.6	35.7	2.7 - 6.1
Osteoporosis	0.9	13.3	1.1 - 1.6
Dyslipidaemia	0.9	1 cohort study	–
Hypertension	4 cases	1 cohort study	–
Peptic ulceration	2 cases	14.3	<1
Gastrointestinal bleed	1 case	1 cohort study	–

*Denominator = total number of participants at risk (n=1652). †Denominator= total number of prospective cohort studies included in review (n=14). ‡Unspecified GICS.

GIDM reported in retrospective studies occurred in 3.0–13.4% of individuals (Table 8). Siagian et al. found that treatment greater than 12 weeks doubled the risk of developing AEs, though not specifically GIDM [85]. Severe cases of GIDM were rare but included two fatalities due to diabetic ketoacidosis [54,93].

**Table 8:** Adverse effects reported in retrospective cohort studies.

Adverse effect	Proportion of participants with AE (%)*	Proportion of retrospective cohort studies reporting AE (%)†	Range of the proportion of participants with AE (%)
Infection	15.4	80.0	8.9 - 53.8
Fungal	4.7	60.0	1.0 - 11.3
Bacterial	2.7	40.0	3.8 - 6.2
Parasitic	1.6	60.0	1.5 - 4.0
Glucocorticoid-induced Cushing syndrome			
Corticosteroid-induced lipodystrophy	2.5	1 cohort study	–
Acneiform eruption	4.1	1 cohort study	–
Weight gain	3.3	40.0	4.6 - 26.9
Skin atrophy	1.2	1 cohort study	–
Hypertension	8.2	80.0	2.0 - 13.8
Glucocorticoid-induced diabetes mellitus	5.6	80.0	3.0 - 23.4
Gastritis	3.7	40.0	5.6 - 10.9
Cataract	1.6	60.0	1.0 - 3.1
Neuropsychiatric effects	1.2	1 cohort study	
Glaucoma	1.2	60.0	1.0 - 2.1
Osteoporosis	1.0	40.0	0.5 - 4.0
Electrolyte disturbance	0.6	1 cohort study	
Peptic ulceration	2 cases	1 cohort study	

*Denominator = total number of participants at risk (n = 496). †Denominator = total number of retrospective cohort studies included in review (n = 5).

71% of GIDM occurred in T2R patients in case reports and case series. Two deaths were linked to prolonged (~18 months) corticosteroid therapy caused by infection in individuals with GIDM [85,86].

### Dyslipidaemia

Negera et al. observed elevated low-density lipoprotein and high-density lipoprotein in T2R patients before and after corticosteroid (*p* = 0.0014), suggesting the possibility of dyslipidaemia associated with corticosteroid exposure, though confounded by the use of multi-drug therapy and the inflammation associated with ENL itself [79].

### Hypothalamic-pituitary-adrenal (HPA) axis suppression

HPA axis suppression was reported in eight patients in three case reports, two case series, and a retrospective study. The diagnosis was made by conducting short Synacthen tests and morning cortisol levels [94,95]. Overall, 75% of individuals were on high-dose oral prednisolone (40–60 mg daily) and onset ranged from 5 months to 2 years form corticosteroid initiation [85]. One report described delayed growth and puberty in a 13-year-old child after 11 months of prednisolone therapy [85].

### Electrolyte disturbance

Hypokalaemia was reported in one retrospective cohort study and in one case report [85,96]. Siagian et al. reported cases 1–3 years after starting corticosteroids, and exclusively in individuals treated for longer than 12 weeks [85]. One case of hypokalaemia may have been compounded by vomiting and chronic diarrhoea due to strongyloidiasis [96].

### Infection adverse effects

Infection was the second most frequently reported corticosteroid-related AE, affecting 16.8% of individuals experiencing AEs. Fungal infections were the most frequently reported infection (63.3%), followed by tuberculosis (48% of bacterial infections) and strongyloidiasis, which, although less frequently reported, constituted nearly half of parasitic infections. A prospective study reported 1.66 times higher risk of any infection in participants with ENL compared to those with T1R (95% CI 1.30- 2.19; *p* < 0.001) [72]. Most infections occurred in corticosteroid-experienced individuals. Among individuals with an infection, 16.5% had glucocorticoid-induced diabetes. A more detailed risk factor assessment of infections was limited due to a lack of data on affected participants’ co-morbidities.

### Fungal infection

Dermatophyte infection was common, reported in up to 51.5% of participants with ENL in RCTs and comprised 82% of infections in prospective cohort studies with median follow up of 11 years. These cases were generally mild and responsive to topical antifungal therapy, occasional requiring oral treatment such as griseofulvin [71,72]. Evidence from trials comparing corticosteroids and placebo trials did not show a significant difference in fungal infections, though longer corticosteroid exposure (32 weeks compared to 20 weeks) in the TENLEP clinical trial demonstrated a trend toward higher risk (RR 1.35, 98% CI 0.99 -1.84; *p* = 0.058) [53].

Rare opportunistic fungi such as chromoblastomycosis and phaeohyphomycosis were also documented, often occurring in agricultural workers who had prolonged high-dose corticosteroids; with a mean duration of 11 months [97,98]. There were no reports of *Pneumocystis jirovecii* pneumonia.

### Bacterial infection

Bacterial infections, mainly identified in case reports and case series, made up 20.7% of all case reports (S5 Table).

### Tuberculosis

Tuberculosis was a significant complication, responsible for almost 10% of infections in case reports, 7.9% in prospective cohorts and nearly a quarter of reported fatalities (Table 4). Cases typically occurred in individuals on high-dose or prolonged corticosteroid therapy. The onset varied from 2 months to over a year after corticosteroid initiation [72].

### Other bacterial infections

Other severe bacterial infections reported in RCTs included osteomyelitis and infective endophthalmitis [72]. Four cases of nocardiosis were reported in patients who received prolonged courses of corticosteroids, including two cases caused by *Nocardia farcinica* and one by *Nocardia nova* [99–102]. One patient who was corticosteroid naïve developed *Staphylococcus aureus* bacteraemia in the context of newly diagnosed human immunodeficiency virus infection and initiation of antiretroviral therapy [103].

### Strongyloidiasis

Infections reported to be due to *Strongyloides stercoralis* were severe and represented 87.5% of parasitic infections in case reports and caused two deaths (S5 Table). All reports of strongyloidiasis were in corticosteroid experienced individuals (treatment duration 5 months to more than 2 years). One case report of *Strongyloides stercoralis* hyperinfection syndrome occurred five years after corticosteroid initiation despite albendazole prophylaxis [104]. No parasitic infections were reported across the RCTs; however, only 21.1% of RCTs specified the use of prophylactic anti-helminthic agents, most frequently albendazole.

### Gastrointestinal adverse effects

#### Gastritis.

Gastritis was the third most reported AE, affecting 12.6% of individuals, although definitions varied across studies. In T1R, 22.4% of participants developed gastritis. TRIPOD 1 found a 50% increased risk of gastritis with prednisolone 20mg daily over 12 weeks versus placebo (RR 1.50, 95% CI 1.10-1.32; *p* = 0.0124). Although the TENLEP subclinical trial showed no difference between corticosteroid and placebo groups, Lambert et al. found higher-dose prednisolone (60 mg tapered over 20 weeks) was associated with increased risk of gastritis compared to lower-dose regimens (RR 7.37, 95% CI 2.43-22.33; *p* < 0.001) [16]. Among participants with T2R, 20.7% of all participants developed gastritis. An RCT involving participants with ENL found that there was significant increase in risk of gastric pain, with an almost ten times higher risk in prednisolone monotherapy compared to ciclosporin with low-dose prednisolone (RR = 9.79, 95% CI 1.43-66.90; *p* < 0.001) [105]. Four prospective cohort studies, with follow-up duration of 6 months-5 years, reported gastritis in 2.4-8% of individuals; most cases were managed with “antacids” or oral ranitidine. Two retrospective cohort studies conducted in Indonesia and Brazil found rates of 5.6% and 10.9% respectively [86,87] (S4 Table).

#### Peptic ulceration.

Peptic ulceration was less frequent than gastritis. In RCTs for T1R, peptic ulceration occurred to up to 2.44% of participants receiving prednisolone 40 mg/day over 20 weeks. One fatal case of perforated peptic ulcer was reported in a participant with ENL who received at least 40mg/day of prednisolone over 16 weeks in an RCT [66]. A case report described another perforated duodenal ulcer requiring surgical intervention in a prolonged corticosteroid user [106]. Observational cohort studies reported a perforated ulcer after two weeks of corticosteroid therapy and two cases of gastric ulcers reported between one and two years after corticosteroid initiation [85].

#### Gastrointestinal bleeding.

Four cases of gastrointestinal bleeding were reported in RCTs, one of which was fatal [66,72](Table 4).

### Ophthalmological adverse effects

#### Cataract.

A total of 2.3% of all individuals developed cataracts, of whom 79.6% were corticosteroid experienced. Cataract was more frequently documented in case reports and series compared to observational studies or RCTs, reflecting their progressive nature and association with long-term corticosteroid use.

Two RCTs each reported a single case of cataract, but the individuals’ characteristics were not described [55,63]. A total of 5.3% of participants among prospective cohort studies developed cataracts, observed solely in individuals with more than 12 months of corticosteroid exposure [71,72,76]. Affected individuals reported visual clouding and light sensitivity which often affected daily activities [71].

Cataracts were reported in 1-3.1% of individuals in retrospective cohort studies from Brazil, Ethiopia and Indonesia involving 358 patients, of whom 57.5% had ENL [24,85,86]. In a retrospective cohort study in Indonesia, all cases occurred in participants who had received more than 12 weeks of corticosteroid therapy [85]. Methods for cataract assessment were not clearly described; however, as data was extracted from pre-existing medical records, diagnoses were likely based on documented symptomatic presentation. A case series reported mean corticosteroid exposure from 11 months to 2.5 years [80]. Posterior subcapsular cataracts were the most common type of cataract reported and several individuals required surgery [99]. The youngest reported individual with a cataract was 27 years old on self-medicated oral corticosteroid therapy [107].

#### Glaucoma.

Glaucoma was found in 0.2% of all individuals. Corticosteroid-induced glaucoma was infrequent, with eight cases reported. The onset of glaucoma ranged from 32 weeks to 3 years after corticosteroid initiation [54,85]. Mishra et al. described concurrent scleromalacia perforans and glaucoma, highlighting the need for caution to prevent severe complications such as ocular perforation [80].

### Cardiovascular adverse effects

Hypertension was reported in 1.6% of patients, while heart failure was not frequently reported. There were few data on the long-term morbidity and mortality of these adverse effects in individuals with leprosy on prolonged corticosteroid therapy.

Five RCTs of T1R/NFI and three of ENL reported 0.9-10% of participants developing hypertension. Participants were screened for hypertension prior to enrolment. There was no significant association between hypertension and cumulative corticosteroid dose across RCTs, though follow-up durations were short (median of 11.2 months), and only two RCTs explicitly described monitoring of blood pressure and the frequency of it [16,66]. No RCTs comparing corticosteroid with placebo reported new-onset hypertension, and no trial demonstrated a statistically significant increase in hypertensive events with higher cumulative corticosteroid doses.

One prospective cohort study, which conducted monthly blood pressure monitoring over three years, identified hypertension in 4.9% of participants receiving corticosteroids for 6–30 months [77].

Four retrospective cohorts of mostly people with ENL reported hypertension in 2-13.8% of patients, typically after more than 12 weeks of high-dose therapy, equivalent to 0.5-1mg/kg/day of prednisolone [85]. Onset ranged from 1–4 weeks to up to 3 years post-initiation [85]. One RCT reported the use of ‘anti-hypertensives’ in a single patient to manage hypertension associated with corticosteroid use [56]; however no other observational studies or RCTs provided details on the management or resolution of AEs following cessation of corticosteroid therapy.

### Thrombosis

Thrombosis developed in 0.8% of individuals. Among those, 75.6% were deep vein thrombosis (DVT) and 22% had pulmonary embolism (PE), with one case of arterial thrombosis. DVT and PE were counted as separate events, even when occurring in the same patient.

Nearly all affected individuals were treated for ENL, with 93.5% receiving both thalidomide and prednisolone. Only one RCT reported a case of DVT while the remainder were described in case series and reports [63]. Two patients developed both DVT and PE on corticosteroid monotherapy: one after 10 months of prednisolone, and another after an unspecified corticosteroid duration [108,109]. No deaths were attributed to thrombosis. The thrombotic episodes were managed by discontinuing thalidomide and initiating anticoagulation.

### Musculoskeletal adverse effects

Osteoporosis occurred in 0.69% of patients, predominantly in those with prolonged corticosteroid use for ENL. One RCT reported a case of vertebral collapse in an individual with ENL treated with 2mg/kg/day of prednisolone, though no further clinical details were provided [55]. In a prospective study, Sugumaran identified one individual with ENL who developed a hip fracture due to osteoporosis [72]. Affected individuals with osteoporosis had received corticosteroids for 6–12 months, and calcium supplementation was routinely given after six months; bisphosphonates were noted as effective but costly [72].

One retrospective study reported onset of osteoporosis around 30 months after corticosteroid initiation [85]. Case series and reports described osteoporosis in corticosteroid-experienced individuals with severe ENL, including a 36-year-old woman who developed thoracic vertebral collapse after intermittent prednisolone use over 10 years [110]. One case of avascular necrosis of the femoral head was reported in a person with ENL [111].

### Neuropsychiatric adverse effects

Neuropsychiatric AEs, including anxiety, depression, insomnia, and corticosteroid-induced psychosis occurred in 0.6% of individuals. Insomnia developed within 9 weeks of corticosteroid initiation in a retrospective review, and corticosteroid-induced psychosis was reported 4.5 years after starting therapy [85]. No clear link was found between neuropsychiatric risk and cumulative corticosteroid dose.

## Discussion

Corticosteroids remain essential for management of T1R, NFI and ENL, but their AEs including associated mortality in people affected by leprosy are clinically significant and likely underreported. Our review offers a comprehensive synthesis of corticosteroid-related AEs in the management of leprosy. Metabolic complications and infection were the most frequently reported corticosteroid-related AEs, while insidious conditions such as osteoporosis and cataract were less frequent. The findings of this review align with broader corticosteroid AEs reported in other conditions, where infection, metabolic and gastrointestinal complications are well-recognised [31,38]. In contrast, hypertension and cardiovascular events were reported less frequently in individuals with leprosy, although this may reflect limited monitoring or reporting of these AEs.

Tuberculosis emerged as the leading corticosteroid-associated cause of mortality, underscoring the need to exclude active tuberculosis prior to corticosteroid initiation and be vigilant for the reactivation of latent disease [27]. Interestingly no reports of *Pneumocystis jirovecii* pneumonia were identified in this review despite the increased risk of this infection in individuals treated with long-term corticosteroid in other conditions [112]. Prophylaxis for *Pneumocystis jirovecii* is recommended in rheumatological conditions. Recently, Ramírez-Perea and colleagues hypothesized that the dapsone component of MDT might afford individuals with leprosy some protection. They recognized that the duration of leprosy reactions often exceeds that of MDT and suggested prophylaxis should be considered in individuals with leprosy who are not taking dapsone. There is no evidence to support this approach at present [113]. Immunocompromised individuals are at greater risk of varicella zoster virus (VZV) and reactivation of hepatitis B virus, warranting assessment of immunity prior to starting corticosteroids [113–115].

Deaths associated with Strongyloides hyperinfection syndrome illustrate the importance of effective prophylaxis for this infestation on starting corticosteroid therapy in individuals at high risk of chronic strongyloidiasis [88,89]. Diagnosis can be challenging as *Strongyloides stercoralis* infection can remain asymptomatic for years and peripheral eosinophilia may be absent in 20–30% of infected individuals [116,117]. A study from Nepal of 145 individuals with leprosy identified 18% with *S. stercoralis* DNA in stool, whereas only two individuals had microscopy evidence of a soil-transmitted helminth infection [118]. Corticosteroids and other immunosuppressive drugs may further suppress peripheral eosinophilia and delay detection [119]. The WHO guidelines on preventative chemotherapy for public health control of strongyloidiasis and the National Leprosy Eradication Programme in India both have clear recommendations for empirical treatment before corticosteroid initiation to prevent disseminated strongyloidiasis [120,121].

HPA axis suppression is difficult to diagnose due to varied presentation. Though rare, the individual reported in the retrospective cohort study of Siagian et al. who suffered from adrenal suppression and growth retardation highlights potential long-term harms [85]. The UK Society for Endocrinology recommends that individuals treated with prednisolone 5mg per day or equivalent for more than 4 weeks should be given a corticosteroid emergency card to warn of adrenal crisis [122]. Advice about “sick day rules” should be provided to prevent adrenal crisis, where an increase in the regular corticosteroid dosage is recommended to reduce the risk of adrenal crisis during intercurrent illness [122]. Adrenal crisis should be suspected in an acutely deteriorating individual on corticosteroids, and may require immediate treatment with intravenous hydrocortisone [123].

Our systematic review has limitations. The review process was conducted primarily by an independent reviewer which may have introduced selection bias. The heterogeneity in study designs, interventions, definitions of AEs, and study quality limited the ability to make direct comparisons between studies and precluded meta-analysis. A further limitation is that we were obliged to rely on the information provided in the authors’ accounts of included studies to ascertain the status of corticosteroid exposure and causes of death. The narrative nature of this review means there is a risk of confounding when exploring the temporal association between corticosteroid exposure and adverse effects. Potential publication bias may have led to the underrepresentation of AEs that are underreported. Most studies were conducted in referral settings, where loss to follow-up may lead to underreporting of the frequency of AEs.

**Fig 2:**
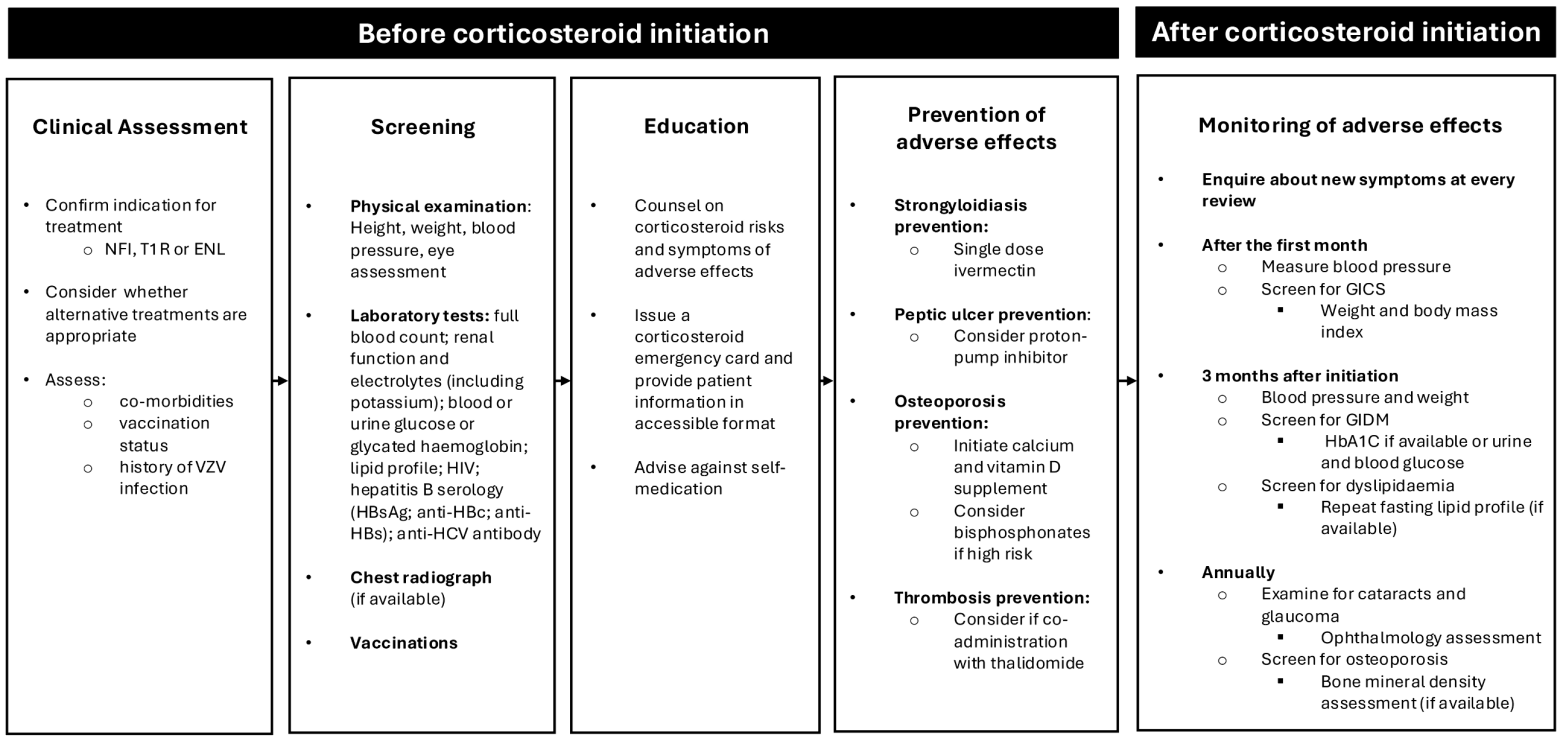
Framework for consideration for the prevention and early detection of adverse effects associated with corticosteroid therapy for the treatment of nerve function impairment, Type 1 reaction or erythema nodosum leprosum in adults. NFI= Nerve function impairment; T1R= Type 1 Reaction; ENL= Erythema nodosum leprosum; VZV= Varicella zoster virus; HIV= Human immunodeficiency virus; HbA1C= Glycated haemoglobin; HBsAg= Hepatitis B surface antigen; Anti-HBc= Hepatitis B Core Antibody; Anti-HBs= Hepatitis B surface antibody; HCV= Hepatitis C Virus; GICS= Glucocorticoid-induced Cushing syndrome; GIDM= Glucocorticoid-induced diabetes mellitus.

Monitoring for AEs is essential for all individuals with leprosy on corticosteroids. Fig 2 provides a framework for consideration when designing context-appropriate monitoring guidelines to identify and reduce the burden of AEs in adults with leprosy in endemic areas [124–126]. Children may need additional monitoring as approximately one-fifth of children exposed to long term oral corticosteroids may experience growth retardation [85,114].

Before corticosteroid initiation, consideration should be given to the availability of corticosteroid-sparing agents, such as thalidomide for ENL [27]. Although the United States National Hansen’s Disease Program (NHDP) recommends methotrexate as an adjunct to corticosteroids for T1R, there remains limited published evidence to support this [127].

Once corticosteroid therapy is indicated, a pre-treatment assessment should screen individuals for metabolic comorbidities, blood-borne viruses (hepatitis B and C, and HIV) and tuberculosis [128]. Where available, a chest radiograph may be used in addition to symptom screening for active tuberculosis in high-risk groups [129]. Routine vaccination status should be assessed and any additional vaccinations considered [130].

Patients should receive appropriate counselling on the risks of corticosteroid treatment before initiation, as emphasized in *Protocolo Clínico e Diretrizes Terapêuticas da Hanseníase* of the Brazilian Ministry of Health [131]. There were several reports of severe adverse outcomes associated with the unsupervised self-administration of corticosteroids found in this review and this should be discouraged [80,106]. Information on AE symptoms and the risks of self-medication without supervision should be provided in accessible formats that account for illiteracy [8].

Prior to initiation of corticosteroids prevention therapies for strongyloidiasis, peptic ulceration, osteoporosis and venous thromboembolism should be considered.

Empirical treatment for strongyloidiasis is essential, with single-dose ivermectin recommended by WHO and The World Gastroenterology Organisation [120,132]. A Cochrane systematic review and meta-analysis found higher cure rates for ivermectin compared to albendazole, with no significant difference in AE rates between the two drugs [133].

Prophylaxis for gastrointestinal disorders was inconsistently reported across studies. The WHO Technical Guidance for reactions and the *Protocolo Clínico e Diretrizes Terapêuticas da Hanseníase* both acknowledge AEs of dyspepsia and gastrointestinal ulceration from corticosteroid use but neither addressed the role of proton pump inhibitors (PPI). The National Institute for Health and Care Excellence (NICE) and EULAR advise considering PPIs for individuals at high risk of gastrointestinal bleeding or dyspepsia [124,134]. In contrast, the Asociación Mexicana de Gastroenterología (AMG) advises against chronic PPI prescription in patients receiving corticosteroids alone, recommending it only when non-steroidal anti-inflammatory drugs or antiplatelet agents are also prescribed [135].

Osteoporosis was often only detected at advanced stages when fractures had occurred [55,72]. Preventive measures with calcium and vitamin D supplementation should be prescribed, and bisphosphonates are recommended for individuals at moderate to high risk of fracture; based on bone mineral density and Fracture Risk Assessment Tool (FRAX) score [33,136]. This is in accordance with Indian Association of Dermatologists, Venereologists and Leprologists (IADVL), American College of Rheumatology, and NICE guidelines [124,136,137].

Thromboprophylaxis should be considered when thalidomide is co-prescribed with corticosteroids as there is an increased risk of thrombosis [138]. The European Myeloma Network guidelines and The Leprosy Mission Trust India (TLMTI) guidance recommend low-dose aspirin for individuals at low risk [125,139]. For patients with multiple risk factors, the European Myeloma Network advises low molecular weight heparin or warfarin [139].

Patients should be asked about symptoms of adverse effects and regularly monitored for hypertension, glucocorticoid-induced diabetes mellitus and dyslipidaemia [140,141]. TLMTI guidance recommends monitoring of blood pressure at every outpatient visit, and glucose after one month and three monthly thereafter [125]. The IADVL manual recommends blood sugar monitoring 24–48 hours after initiation of corticosteroids, and no further monitoring if levels are normal [137].

The incidence of cataract was largely associated with prolonged exposure [142,143], and since individuals with leprosy are already predisposed to ocular complications, frequent monitoring and early detection of corticosteroid-associated cataract is essential to prevent G2D [143]. Ophthalmic monitoring should be standardized to detect early cataract and glaucoma, as the young onset of cataracts reported in case reports highlight the danger of unsupervised high-dose corticosteroids [80,125]. Both TLMTI and IADVL suggest ophthalmological evaluation, with monitoring frequency ranging from every month to every 6–12 months [125,137].

Effective corticosteroid stewardship requires the lowest effective dose for the shortest possible duration. Corticosteroid stewardship is critical but may be constrained by limited resources in many leprosy endemic settings [144]. Effective monitoring imposes financial and logistical burdens on the health system and on patients. The direct and indirect costs to affected individuals of attendance at leprosy referral centres may make visits for regular monitoring challenging [145]. Counselling and peer-support interventions may have a role in enhancing corticosteroid stewardship [10,146]. WHO-aligned pharmacovigilance and surveillance strategies to support corticosteroid AE reporting and monitoring could help reduce avoidable morbidity and mortality [147].

Reducing AEs associated with corticosteroids in people affected by leprosy is a priority. This requires patient education, thorough clinical assessment prior to initiation, robust monitoring, effective morbidity prevention and access to effective alternative treatments for leprosy reactions. The WHO has recognised the need for improved pharmacovigilance for MDT; this also needs to be strengthened for the adverse effects of corticosteroids and other immunomodulatory treatments in the management of leprosy reactions [27].

## Supporting information

S1 File PRISMA 2020 checklist from Page MJ, McKenzie JE, Bossuyt PM, Boutron I, Hoffmann TC, Mulrow CD, et al. The PRISMA 2020 statement: an updated guideline for reporting systematic reviews. BMJ 2021;372:n71. https://doi.org/10.1136/bmj.n71, licensed under CC BY 4.0.
(PDF)

S1 Text Search strategy. (PDF)

S1 Table Risk of bias assessment for randomised controlled trials. (PDF)

S2 Table Risk of bias assessment for observational studies. (PDF)

S3 Table Table of summary of prospective cohort studies. (PDF)

S4 Table Table of summary of retrospective cohort studies. (PDF)

S5 Table Proportion of case reports and case series recording rarer adverse events. (PDF)
