# Supplementary material for: A systematic review of adverse effects associated with systemic corticosteroids in the management of leprosy
Source: PLoS Negl Trop Dis. 2026 Mar 26;20(3):e0014152. doi: 10.1371/journal.pntd.0014152 (PMC13038111; doi:10.1371/journal.pntd.0014152)
Supplement: S1 Text — (PDF) [file pntd.0014152.s002.pdf]

S1 Text: Search Strategy.

1. exp Leprosy
2. (lepros* or Hansen* or leprom* or mycobacterium lepr*).mp.
3. 1 or 2
4. (corticosteroid or steroid or prednisolone or prednisone or dexamethasone or methylprednisolone).mp.
5. exp Steroids/
6. exp Adrenal Cortex Hormones/
7. 4 or 5 or 6
8. exp "Drug-Related Side Effects and Adverse Reactions"/
9. exp product surveillance, postmarketing/ or exp adverse drug reaction reporting systems/ or exp clinical trials, phase iv as topic/ or exp pharmacovigilance/
10. exp Drug Hypersensitivity/
11. exp Long Term Adverse Effects/
12. exp Safety-Based Drug Withdrawals/
13. exp Drug Recalls/
14. exp Abnormalities, Drug-Induced/
15. (safe* or adverse* or undesirable or harm* or injurious or risk or risks or reaction* or complication* or poison*).ti,kf.
16. (side effect* or safety or unsafe).ti,ab,kf.
17. ((adverse or undesirable or harm* or toxic or injurious or serious or fatal) adj3 (effect* or reaction* or event* or outcome* or incident*)).ab.
18. ((drug or chemically) adj induced).ti,ab,kf.
19. (toxic or toxicit* or toxologic* or intoxication or noxious or tolerability or teratogen*).ti,ab,kf.
20. (warning* or recall* or withdrawn* or withdrawal*).ti,kf.
21. (death or deaths or fatal or fatality or fatalities).ti,kf.
22. or/8-21
23. (react* or reversal reaction or complicat* or erythema nodosum leprosum or ENL or neuritis or neuropathy or inflam* or nerve function impair*).mp.
24. (“Type 1” or “Type one” or “Type 2” or “Type two”) adj1 reaction
25. 23 or 24
26. 3 and 7 and 22 and 25
