# Supplementary material for: A systematic review of adverse effects associated with systemic corticosteroids in the management of leprosy
Source: PLoS Negl Trop Dis. 2026 Mar 26;20(3):e0014152. doi: 10.1371/journal.pntd.0014152 (PMC13038111; doi:10.1371/journal.pntd.0014152)
Supplement: S1 Table — (PDF) [file pntd.0014152.s003.pdf]

S1 Table: Risk of Bias Assessment for Randomised Controlled Trials

The Cochrane Risk-of-Bias Tool (version 2): Randomised Controlled Trials (Prophylactic, T1R and NFI)

Study ID	Randomisation process	Deviations from the intended interventions	Missing outcome data	Measurement of the outcome	Selection of the reported result	Overall
Doull et al 1967	!	!	!	+	!	!
Garbino et al 2008	!	+	+	+	!	!
Lambert et al 2016b	+	+	+	+	!	+
Lockwood et al 2017	+	+	!	+	+	+
Marlowe et al 2004	+	+	+	!	!	!
Richardus et al 2003a	+	+	+	+	!	+
Smith et al 2004	+	+	+	+	!	+
Van Brakel et al 2003	+	+	+	+	!	+
Wagenaar et al 2017	+	+	+	+	+	+
Walker et al 2011	+	+	+	+	!	+

\+ Low risk ! Some concerns - High risk

The Cochrane Risk-of-Bias Tool (version 2): Randomised Controlled Trials (T2R)

Study ID	Randomisation process	Deviations from the intended interventions	Missing outcome data	Measurement of the outcome	Selection of the reported result	Overall
Girdhar et al 2002	!	!	!	-	!	-
Hanumanthu et al 2021	!	!	+	!	!	!
Ing et al 1969	!	!	!	!	!	!
Kar et al 2015	!	-	!	-	-	-
Karat et al 1969	!	!	!	+	!	!
Karat et al 1970	+	!	+	+	!	+
Kaur et al 2009	+	!	+	!	!	!
Lambert et al 2016a	+	+	+	+	!	+
Martinus et al 2020	!	+	+	+	!	+
Roy et al 2015	+	+	+	+	!	+
Sakhare et al 2024	!	-	!	-	!	-

+ Low risk   ! Some concerns   - High risk
