# Supplementary material for: A systematic review of adverse effects associated with systemic corticosteroids in the management of leprosy
Source: PLoS Negl Trop Dis. 2026 Mar 26;20(3):e0014152. doi: 10.1371/journal.pntd.0014152 (PMC13038111; doi:10.1371/journal.pntd.0014152)
Supplement: S2 Table — (PDF) [file pntd.0014152.s004.pdf]

***S2 Table**: Risk of Bias Assessment for Observational Studies*

Newcastle-Ottawa Quality Assessment Scale: Observational Studies

Study ID	Selection	Comparability	Outcome	Total Score	Risk of Bias
**Bandeira et al 2019**	★★★	★	★	5	Unclear
**Barthula et al 2023**	★★★		★★★	6	Unclear
**Hanumanthu et al 2021**	★★★★	★★	★★★	9	Low
**Hossain et al 2012**	★★★		★★★	6	Unclear
**Kiran et al 1985**	★★★		★★★	6	Unclear
**Kundu et al 1982**	★★★		★★★	6	Unclear
**Lambert et al 2016a**	★★★★	★★	★★★	9	Low
**Lambert et al 2016b**	★★★★	★★	★★★	9	Low
**Listiyawati et al 2015**	★★		★★	5	Unclear
**Lockwood et al 2017**	★★★★	★★	★★★	9	Low
**Marlowe et al 2004**	★★★★	★	★★★	8	Low
**Mishra et al 2020**	★★★		★★★	6	Unclear
**Nabarro et al 2016**	★★★		★★	5	Unclear
**Neves et al 2019**	★★★		★★	5	Unclear
**Papang et al 2009**	★★★		★★★	6	Unclear
**Quyum et al 2020**	★★★★	★★	★★	8	Low

**Rahul et al 2015**	★★★★	★★	★★★	9	Low
**Roy et al 2015**	★★★	★★	★★	7	Low
**Sakhare et al 2024**	★★★		★★	5	Unclear
**Saunderson et al 2000**	★★★		★★★	6	Unclear
**Shetty et al 2010**	★★★★	★★	★★★	9	Low
**Siagian et al 2022**	★★		★★	4	Unclear
**Singla et al 2020**	★★★		★★★	6	Unclear
**Srinivasan et al 1982**	★★★		★★★	6	Unclear
**Sugumaran 1997**	★★★		★★★	6	Unclear
**Sugumaran 1998**	★★★		★★★	6	Unclear
**Thirugnanam et al 1985**	★★★		★★★	6	Unclear
**Walker et al 2014**	★★★		★★	5	Unclear
