# Supplementary material for: A systematic review of adverse effects associated with systemic corticosteroids in the management of leprosy
Source: PLoS Negl Trop Dis. 2026 Mar 26;20(3):e0014152. doi: 10.1371/journal.pntd.0014152 (PMC13038111; doi:10.1371/journal.pntd.0014152)
Supplement: S3 Table — (PDF) [file pntd.0014152.s005.pdf]

S3 Table: Summary of Prospective Cohort Studies.

Study ID	Sample Size	No. of patients at risk [Male%]	Type and Dose of Corticosteroid	Duration of corticosteroid therapy	Duration of Follow-Up (months)	Co-interventions for reaction	Adverse events per 100 patients	Risk of Bias (NOS)
**Type 1 Reactions and Nerve function impairment and Erythema nodosum leprosum**								
**Kundu et al 1982 (70)**	30	30 [93.3%]	PO Betamethasone 60mg/day (tapered)	3 weeks	6	None	213.3	🟧
**Sugumaran 1997 (71)**	149	149 [85.9%]	PO Prednisolone 60mg/day (tapered)	5 months	60	None	195.3	🟧
**Sugumaran 1998 (72)**	830	830 [N/A]	PO Prednisolone 40-60 mg/day (tapered)	1-12 months	12	None	156.5	🟧
**Papang et al 2009 (73)**	81	81 [N/A]	PO Prednisolone 30mg/day (tapered)	Varied	3	None	34.6	🟧
**Srinivasan et al 1982 (74)**	25	25 [100%]	PO Prednisolone 60mg (tapered)	20 weeks	24	None	8.0	🟧
**Thirugnanam et al 1985 (75)**	34	34 [N/A]	PO Prednisolone (dosage unavailable)	14-436 days	3	Clofazimine	5.9	🟧
**Saunderson et al 2000 (76)**	650	185 [64%]	PO Prednisolone (dosage unavailable)	Unclear	132	None	1.0	🟧
**Erythema nodosum leprosum only**								
**Singla et al 2020 (77)**	134	82 [74.6%]	PO Prednisolone 1mg/kg/day (tapered)	Unclear	36	None	101.2	🟧
**Hossain et al 2012 (78)**	9	9 [77.8%]	PO Prednisolone 30-40mg/day (tapered)	3-6 months	60	None	66.7	🟧

**Negera et al 2018 (79)**	60	30<br><br>[70%]	PO Prednisolone<br>40mg/day<br>(tapered)	24 weeks	24	None	50.0	🟩
**Mishra et al 2022 (84)**	30	30<br><br>[83%]	PO Prednisolone<br>40mg/day<br>(tapered)	Unclear	6	Thalidomide<br>Clofazimine	16.7	🟧
**Unquantified**								
**Bandeira et al 2019 (81)**	34	34<br><br>[58.8%]	PO Prednisolone<br><br>(Dosage unavailable)	Unclear	18	None	N/A	🟧
**Shetty et al 2010 (82)**	200	100<br><br>[80.0%]	PO Prednisolone<br>40mg/day<br>(tapered)	Unclear	18	None	N/A	🟩
**Kiran et al 1985 (83)**	33	33	PO Prednisolone<br>25mg/day<br>(tapered)	5 months	6-13	None	N/A	🟧

N/A= Not available
NOS= Newcastle-Ottawa Scale
