# Supplementary material for: A systematic review of adverse effects associated with systemic corticosteroids in the management of leprosy
Source: PLoS Negl Trop Dis. 2026 Mar 26;20(3):e0014152. doi: 10.1371/journal.pntd.0014152 (PMC13038111; doi:10.1371/journal.pntd.0014152)
Supplement: S4 Table — (PDF) [file pntd.0014152.s006.pdf]

S4 Table: Summary of Retrospective Cohort Studies.

Study ID	Sample Size	Reaction type	No. of patients at risk [Male %]	Type & Dose of corticosteroid	Duration of corticosteroid therapy	Co-interventions for reactions	Adverse events per 100 patients	Risk of Bias (NOS)
**Siagian et al 2022 (85)**	195	T1R & T2R	195 [N/A]	Equivalent dosages of 0.5-1mg/kg/day Prednisolone (29.2%), Methylprednisolone (70.3%), Dexamethasone (0.5%)	12 weeks to 6 years	Not reported	117.4	🟧
**Walker et al 2014 (24)**	99	T2R	99 [62.0%]	PO Prednisolone 60mg/day (tapered)	Range <6 to >48 months	Clofazimine, (61.2%) Chloroquine (6.1%), Methotrexate (1 case), Ciclosporin (1 case)	42.4	🟧
**Neves et al 2019 (86)**	112	T2R	112 [72.0%]	PO Prednisolone (N/A)	Median duration 35 months	Thalidomide (65%)	27.8	🟧
**Nabarro et al 2016 (21)**	30	T2R	26 [66.7%]	PO Prednisolone (Ranged from 5-80mg/day)	Median duration 9 months	Clofazimine, Thalidomide, Azathioprine	56.7	🟧
**Listiyawati et al 2015 (87)**	112	T2R	112 [74.1%]	PO Methylprednisolone (N/A)	Unclear	Clofazimine, NSAIDs	8.9	🟧

N/A=Not available
NOS=Newcastle-Ottawa Scale
