# Supplementary material for: A systematic review of adverse effects associated with systemic corticosteroids in the management of leprosy
Source: PLoS Negl Trop Dis. 2026 Mar 26;20(3):e0014152. doi: 10.1371/journal.pntd.0014152 (PMC13038111; doi:10.1371/journal.pntd.0014152)
Supplement: S5 Table — (PDF) [file pntd.0014152.s007.pdf]

S5 Table: Proportion of case reports and case series recording rarer adverse events

Adverse event	Proportion of case reports reporting AE (%)*	Proportion of case series reporting AE (%)†
**Infection**	58.6	38.5
**Bacterial**	20.7	3.4
**Tuberculosis**	5.2	3.4
**Fungal**	19.0	0
**Endemic mycoses**	5.2	0
**Parasites**	13.8	1 case series
**Strongyloidiasis**	12.1	0
**Thrombosis**	12.1	30.8
**DVT**	12.1	23.1
**PE**	1 case report	15.4
**Osteoporosis**	6.9	30.8
**HPA suppression**	5.2	0
**Perforated ulcer**	1 case report	0
**Glaucoma**	0	1 case series
**Avascular necrosis**	0	1 case series

*Denominator= total number of case reports (n=58)
†Denominator = total number of case series (n=13)
